# A Validated Method for the Simultaneous Determination of Methamphetamine and 3,4-Methylenedioxy-*N*-methamphetamine in Blood-Based Liquid Chromatography-Tandem Mass Spectrometry

**DOI:** 10.1155/2020/8862679

**Published:** 2020-11-26

**Authors:** Dinh-Vu Le, Trong-Tuan Nguyen, Van-Trong Nguyen

**Affiliations:** ^1^Faculty of Chemical Engineering, Industrial University of Ho Chi Minh City, 12 Nguyen Van Bao Str. Go Vap, Ho Chi Minh City 70000, Vietnam; ^2^Forensic Medicine Centre of Ho Chi Minh City, 336 Tran Phu. District 5, Ho Chi Minh City 70000, Vietnam

## Abstract

A liquid chromatography-tandem mass spectrometry (HPLC-MS/MS) method has been validated for the simultaneous determination of methamphetamine (MA) and 3,4-methylenedioxy-N-methamphetamine (MDMA) in the blood sample. Under the optimal experimental conditions, the concentration of MA can be determined in the range from 1 *µ*g/L to 5000 *µ*g/L with the method detection limit (MDL) of 0.31 *µ*g/L. The range from 0.5 to 500 *µ*g/L is observed for the determination of MDMA with the MDL down to 0.25 *µ*g/L. The practical applicability of the method is performed with the recovery ranging from 85.3% to 94% for MA and from 86.9% to 95.5% for MDMA. At the different concentrations of drugs, the relative standard deviations (RSD) for both MA and MDMA are lower than 5.7%. The method was applied to analyse 1995 blood samples that had been collected from the Forensic Medicine Centre of Ho Chi Minh City. The results showed 1.75% positive with MA and 0.25% positive with MDMA. These two drugs take 10% of the total drugs positive samples. By using deuterium-labelled methamphetamine-d5 and 3,4-methylenedioxy-N-methamphetamine-d5 as the internal standards in the determination and the use of MS/MS in multiple reaction monitoring mode signal readout, the method exhibits robustness specificity and can be applied in simultaneous determination of MA and MDMA in blood with high selectivity and sensitivity.

## 1. Introduction

In recent decades, the illicit use of methamphetamine, 3,4-methylenedioxy-N-methamphetamine, and other psychostimulant drugs has dramatically increased in many countries [1]. Methamphetamine and 3,4-methylenedioxy-N-methamphetamine are amphetamine-type stimulants. They are abuse drugs with stimulant or hallucinogenic properties [2, 3]. Among amphetamine-type stimulants, MA is known to be a powerful stimulant of the central nervous system and the most frequently abused drug. MA is named as meth, blue, ice, and crystal, among many other terms, and it takes the form of a white, odorless, bitter-tasting crystalline powder that easily dissolves in water or alcohol. MA exposure is associated with neurochemical and structural alterations in areas of the brain known to affect attention and behaviour [4, 5]. Prenatal MA exposure has shown deleterious MA-induced effects on both the mother and offspring. In addition to structural eye defects, delayed motor development, and learning impairments [6, 7], another amphetamine-type stimulant is MDMA, which is widely used as a recreational drug among young people. MDMA has been shown to reduce the phenotypic expression of 5-HT throughout the adult brain [8, 9]. Some evidence indicates the phenotype loss for MDMA-induced serotonin neurotoxicity in adults [10, 11].

For diagnosis and therapy to the person who is drug-addicted, assessment MA and MDMA level in blood is an indispensable step. Gas chromatography-tandem mass spectrometry (GC/MS) had been used for the analysis of these drugs for many years [12, 13], but it sometimes suffers challenging to apply to thermally labile drugs as well as polar, nonvolatile drugs without derivatization. Recently, high-performance liquid chromatography-tandem mass spectrometry has been chosen for the determination of MA, MDMA, and other stimulants, but these methods are only used for the analysis of drug products, urine samples [14, 15]. The HPLC method using matrix-assisted laser desorption ionization-time of flight mass spectrometry was also validated for the analysis of MA and MDMA in blood samples [16]. Although a very small sample is needed (20 *µ*L), this method still suffers from some drawbacks such as multistep in sample preparation and the narrow range in quantitation (0.003–0.050 mg/L). With the toxic blood concentrations of MA and MDMA ranging up to 40 mg/L for MA and 0.5 mg/L for MDMA, this method cannot be applied to evaluate and classify the effect level of these drugs in the blood sample (therapeutic, toxic, and comatose-fatal level) [17–19]. Focusing on this point, this study aims to validate a sensitive method for the simultaneous determination of MA and MDMA in the blood sample by HPLC-MS/MS for further application to the analysis of these drugs in the real sample. By using deuterium-labelled MA-d5 and MDMA-d5 as the internal standards and the use of MS/MS in multiple reaction monitoring mode signal readout, the method promises high specificity and sensitivity in the analysis. Besides, by using the real blood sample in the validation, the merits of the method can be obtained in high confidence and can be applied for diagnostic, therapeutic, or drug toxicology.

## 2. Experimental

### 2.1. Materials and Reagents

All reagents used in this work were of analytical grade. Methamphetamine 1 mg/mL in methanol, 3,4-methylenedioxy-N-methamphetamine 1 mg/mL in methanol, methamphetamine-d5 1 mg/mL in methanol, and 3,4-methylenedioxy-N-methamphetamine-d5 1 mg/mL in methanol were purchased from Sigma-Aldrich (Singapore). Acetonitrile (ACN), methanol, acetone, dichloromethane, 2-propanol, NH_4_OH 25%, KH_2_PO_4_, Na_2_HPO_4_, and LiChrolut® RP-18 (40–63 *µ*m) 500 mg 3 ml standard PP-tubes were purchased from Merck (Merck, Darmstadt, Germany). All solutions were prepared and diluted using ultrapure water (with an electric resistivity >18.3 MΩ cm) produced by a Millipore Milli Q system (Billerica, MA, USA). Standard and internal standard working solutions (100 mg/L) were prepared from the stock standard solution (1000 mg/L), and these solutions were stored at 4°C for use in 3 months.

### 2.2. HPLC-MS/MS Analysis

Blood samples were prepared according to the previous reports [20, 21], briefly described as follows: 2 mL blood sample was added with 250 *µ*L MA-d5 2 mg/L and 250 *µ*L MDMA-d5 2 mg/L into a 15 mL centrifuge tube, and this mixture was vortexed for 15 minutes and then left for 10 minutes. After centrifuging the mixture at 8000 rpm for 5 minutes, the analytes in the supernatant were cleaned by C18 extraction column in four steps: (1) the column is activated by 1 mL methanol, 1 mL distilled water, and 1 mL phosphate buffer solution (pH = 6); (2) 1 mL of the prepared sample is loaded within 1 minute; (3) the column is washed with 5 ml distilled water/acetone (20 : 80, v/v), waiting for rinse solution completely running out of the column in 5 min; and (4) the analytes are eluted by 1 mL methanol. The obtained solution was evaporated and rediluted in 500 *μ*L MeOH and then filtered through a 0.45 *μ*m filter paper. 5 *µ*L final solution was analysed by HPLC-MS/MS under operating conditions shown in Table 1.

## 3. Results and Discussion

### 3.1. The Specificity of the Method

In order to confirm the accuracy in the determination of MA and MDMA in blood, the specificity, linearity, method detection limit (MDL), accuracy, and precision were evaluated according to the guidance in ASB Standard 036–2017 and ANSI/ASB Standard 072–2019 [22, 23].

**Figure 1:**
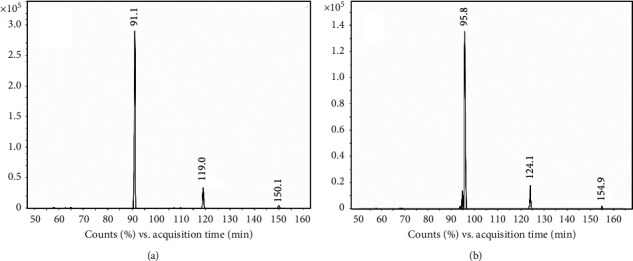
Characterization of analytes by HPLC-MS/MS: the highest signal ion of MA was 91.1 (a), and the highest signal ion of MA-d5 was 95.8 (b).

**Figure 2:**
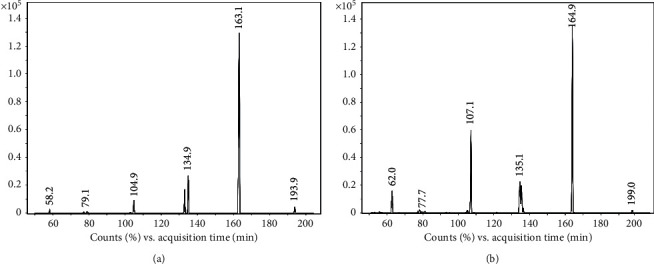
Characterization of analytes by HPLC-MS/MS: the highest signal ion of MDMA was 163.1 (a), and the highest signal ion of MDMA-d5 was 164.9 (b).

The characterization of MA and MDMA was verified by analysis of the standards. The chromatography spectrum was observed in multiple reaction monitoring modes (MRM) with mass fragments shown in Figures 1 and 2. The retention time, precursor, quantification, and confirmation fragment are presented in Table 2.

**Figure 3:**
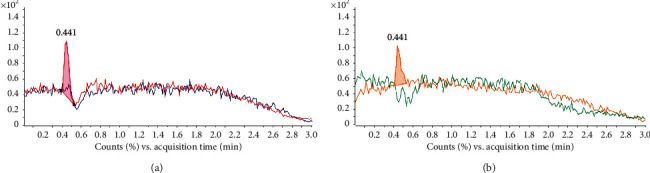
The specificity of MA analysis by HPLC-MS/MS. (a) The quantification ion (150 > 91) chromatograms for blank matrix (dark blue) and spiked 0.3 *μ*g/L MA sample (red). (b) The confirmation ion (150 > 119) chromatogram for blank matrix (green) and spiked 0.3 *μ*g/L MDMA sample (yellow).

**Figure 4:**
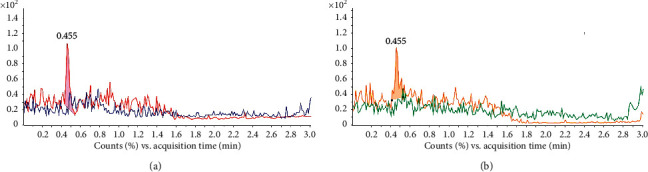
The specificity of the MDMA analysis by HPLC-MS/MS. (a) The quantification ion (194 > 163) chromatograms for blank matrix (dark blue) and spiked 0.3 *μ*g/L MDMA sample (red); (b) The confirmation ion (194 > 105) chromatograms for blank matrix (green) and spiked 0.3 *μ*g/L MDMA sample (yellow).

The specificity of MA and MDMA detection was verified by analysis of the blank sample and spiked samples. MRM chromatograms of product fragments for each analyte were observed to confirm the method specificity. The blank sample chromatograms showed that there was not any signal at the retention time from 0 to 3 min for quantification ion (150 > 91). In contrast, when spiking 0.2 *µ*g/L MA to blank sample, the single peak appeared at ∼0.441 min (Figure 3(a)). A similar result was also obtained for confirmation ion (150 > 119) (Figure 3(b)). This confirmed that there was not any interference signal of the blank matrix to MA detection. The chromatograms of qualification fragment (194 > 105) and confirmation fragment (194 > 163) for MDMA were recorded. There was not a peak for the blank matrix, but it was obviously changed with the spiked 0.2 *µ*g/L MDMA sample (Figure 4). All these results confirmed the high specificity of the method for detecting both MA and MDMA in blood sample.

### 3.2. The Linearity of the Method

The matrix-matched calibration was used in order to minimize the matrix effect to the analytical signal. The blank matrix was simultaneously spiked with proper amounts of MA and MDMA, 250 *µ*L MA-d5 2 mg/L and 250 *µ*L MDMA-d5 2 mg/L before extracted and analysed according to Section 2.2. The calibration curves were the correlation between the ratios of peak area for each standard of analyte (sd) to the internal standard (isd) with a rate of concentration. As shown in Table 3, MA in a blood sample can be determined in the range from 1 *µ*g/L to 5000 *µ*g/L with the coefficient correlation (*R*^2^) of 0.998. The concentration that can be analysed for MDMA is in a range from 0.5 to 500 *µ*g/L with *R*^2^ ～0.999. This is an extensive range, and a low concentration of MA and MDMA in the blood can be determined. The stability of the linearity is also observed in three different days. The coefficient correlation for both MA and MDMA is obtained stable from 0.998 to 0.999. These results demonstrate that both calibration curves are available for the determination of MA and MDMA in the blood sample.

### 3.3. Method Detection Limit and Limit of Quantitation

Method detection and quantitation limit for the determination of MA and MDMA in blood were evaluated based on the analysis of the spiked sample. Eleven repetitive experiments for three spiked samples were carried out. The blank samples were used to be negative with MA and MDMA. MDL of the method was calculated as the three times of standard deviation (SD), and the MQL was as ten times of SD. MDL for MA determination was lower than of 0.31 *µ*g/L and was lower than 0.25 *µ*g/L for MDMA (Table 4). At the MDL level, the quantification ion signal's response ratio relative to the confirmation ion signal was 0.43 for MA and 0.51 for MDMA. These values were unity with the calibration range level that confirmed the obtained MDL to be corrected. This is 10 folds more sensitive than previously reported [16]. Although a little complex sample preparation, the result showed that the method is very sensitive for the determination of MA and MDMA in blood samples.

### 3.4. Accuracy and Precision of the Method

Extraction recovery was calculated by comparing the signal obtained from MA, MDMA spiked into blood before the extraction with the signal obtained from MA, MDMA spiked into MeOH with the same final volume. The extraction recovery ranges from 89–97% for both MA and MDMA. Due to the lack of certified reference materials, the trueness was evaluated by intralaboratory reproducibility (% recovery). The recovery assay results of samples spiked at three levels: 10, 100, and 300 *µ*g/L MA and MDMA on three different days by two analysts. The recovery of MA and MDMA was from 85.3 to 94% for MA and 86.9 to 95.5% for MDMA (Table 5). The recovery was stable in difference days and analysts with repeatability (RSD) below 5.2%.

The repeatability of the method was also evaluated in six repetitive analyses of three blood samples. The relative standard deviations were 5.2%, 4.5%, and 4.3% at the MA level of 24.8, 102.6, and 281.9 *µ*g/L, respectively. RSD at the MDMA concentration of 17, 137.3, and 314.6 *µ*g/L were 5.7%, 4.3%, and 4.8%, respectively. According to the ABS standard 036–2017, this was much lower than the maximum RSD values acceptable (<20%). Therefore, it can be stated that the method exhibited highly accurate and precision for MA and MDMA determination in blood.

### 3.5. Application of the Method for Determination of MA and MDMA in the Blood Sample

**Figure 5:**
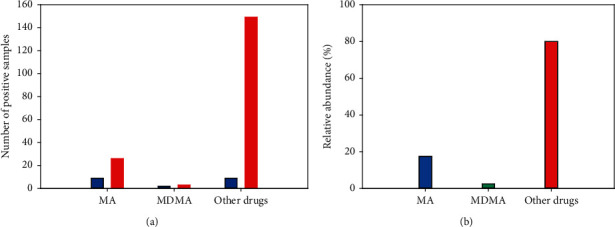
The application of the method for analysis of 1995 human blood samples. (a) The number of positive samples with male (red) and female (blue). (b) The relative abundance of MA (blue), MDMA (green), and other drugs (red) within positive drug samples. Samples were prepared and analysed under the optimal condition, according to Section 2.2.

The method was applied for analysing 1995 blood samples. These samples were collected from the Forensic Medicine Centre of Ho Chi Minh City and were analysed for MA, MDMA, and other drugs such as morphine, codeine, acetaminophen, and ketamine. The percentage of total drugs positive sample was 10.02%, in which MA positive sample was 1.75%, and MDMA was 0.25%. Calculating within each kind of drug, the positive sample from the female was 25.7%, 40%, and 5.6% for MA, MDMA, and other drugs, respectively. The related abundance of MA, MDMA positive blood sample, was also compared within the total drug positive sample. The percentage of MA, MDMA, and other drugs was 17.5%, 2.5%, and 80%, respectively (Figure 5).

The results were also classified in the range of drug concentration that differs impacts on human health. For MA, the range of concentration in therapeutic, toxic, or comatose-fatal was 0.01–0.2 mg/L, 0.2–10 mg/L, and >10 mg/L. The range for MDMA was 0.1–0.35 mg/L, 0.35–0.5 mg/L, and >0.4 mg/L [17–19]. Compared within total MA positive, MA concentration of therapeutic was 55%, and in level of toxic was 45%. No sample observed had the MA level of comatose-fatal effective. The similar results were obtained for MDMA with a percentage of 60% in the therapeutic case and 40% in the toxic case within positive samples.

## 4. Conclusion

In summary, a sensitive method has been validated for the simultaneous determination of MA and MDMA in blood samples based on deuterium-labelled internal standards and liquid chromatography-tandem mass spectrometry. The method exhibits supersensitivity and selective in the determination of MA and MDMA in the blood sample. Based on this method, the calibration curves were obtained in an extensive range and very high correlation coefficient (>0.998) with an extremely low method detection limit (～0.3 *µ*g/L). The desirable precision and accuracy were also obtained with relative standard deviations lower than 5.7%. And the recovery ranges from 85.3 to 95.5%. The practical usefulness of this work was demonstrated by evaluating MA and MDMA in real samples. In 1995 human blood samples, the MA and MDMA positive case was 1.75% and 0.25%, respectively. In comparison with other drugs positive in the blood sample, MA and MDMA occupied 10%; this showed that these drugs were used popularly. Most MA and MDMA positive samples were in the level of therapeutic or toxic, and there was not positive sample in the level of comatose-fatal. Taken together, this work supplies a supersensitive and accurate method for the determination of MA and MDMA in the blood sample.

## Figures and Tables

**Table 1:** Operating conditions of HPLC-MS/MS for determination MA and MDMA in blood.

Instrument parameter	
HPLC-Agilent 1290 Infinity	
Autosampler	Agilent G4226 A
Column	Agilent eclipse plus C18 RRHD analytical column, 2.1 × 50 mm, 1.8 *µ*m (959757–902)
Column temperature	30°C
Flow rate	0.3 mL/min
Mobile phase	Water contains 0.1% formic acid (A) and acetonitrile (B)
Gradient solvent (%B, min)	Initial, 80%; 1 min, 80%; 2.2 min, 100%; 2.8 min, 80%
Injection volume	5 *µ*L

MS-Agilent 6490 Triple Quad	
Capillary (vcap)	4000 V (positive)
Gas temperature	350°C
Gas flow	He, 11 L/min
Nebulizer	35 psi
Nozzle voltage	1500 V (positive)
RF	110–200 V (positive)

**Table 2:** Characterization of analytes by GC-MS/MS.

Analytes	Retention time (min)	Scan time (s)	Precursor ion (m/*z*)	MRM transition, m/*z* (collision energy, V)	
				Quantification	Confirmation
Methamphetamine	0.414–0.478	0.8	150	150 > 91(13)	150 > 119 (5)
3,4-Methylenedioxy-N-methamphetamine	0.401–0.469	0.7	194	194 > 163 (9)	194 > 105 (25)
Methamphetamine–d5	0.407–0.476	0.8	155	155 > 96 (13)	155 > 124 (9)
3,4-Methylenedioxy-*N*-methamphetamine-d5	0.409–0.435	0.7	199	199 > 165 (9)	199 > 107 (21)

**Table 3:** The linearity for determination of MA and MDMA in the blood sample.

Time	Concentration range (*µ*g/L)	Area_sd_/area_isd_ = slope^*∗*^(sd)/(isd) + intercept		Correlation coefficient (*R*^2^)
		Slope	Intercept	
MA				
Day 1	1–5000	0. 5458	0.6567	0.998
Day 2	1–5000	0.5533	0.5018	0.999
Day 3	1–5000	0.5529	0.7721	0.999

MDMA				
Day 1	0.5–500	0.2984	0.0563	0.999
Day 2	0.5–500	0.3062	0.0352	0.999
Day 3	0.5–500	0.3046	0.0256	0.999

**Table 4:** The method detection and quantitation limit of MA and MDMA in blood.

Sample	Spiked (*µ*g/L)	Mean (*µ*g/L)	SD (*µ*g/L)	MDL (*µ*g/L)	MQL (*µ*g/L)
MA					
Sample 1	0.9	0.84	0.06	0.19	0.64
Sample 2	1.2	1.04	0.08	0.24	0.81
Sample 3	1.5	1.25	0.10	0.31	1.00

MDMA					
Sample 1	0.8	0.81	0.05	0.15	0.5
Sample 2	1.0	0.87	0.07	0.21	0.68
Sample 3	1.2	1.05	0.08	0.25	0.84

**Table 5:** Method accuracy of determination of MA and MDMA in the blood sample.

Sample	Standard spiked (*µ*g/L)	Initial sample concentration (*µ*g/L)	Spiked sample concentration (*µ*g/L)	Calculated concentration (*µ*g/L)	Average recovery (%, *n* *=* *6*)
MA					
Sample 1	10	10	24.8	8.53	85.3
Sample 2	100	100	102.6	93.8	93.8
Sample 3	300	32.7	314.6	281.9	94.0

MDMA					
Sample 1	10	8.7	17	8.6	86.9
Sample 2	100	46	137.3	91.3	91.3
Sample 3	300	68.4	354.9	286.5	95.5

## Data Availability

The data used to support the findings of this study are included within the article.
